# Digitalisierung und Künstliche Intelligenz in der Intensiv- und Notfallmedizin: Impulspapier der Deutschen Gesellschaft für Internistische Intensivmedizin und Notfallmedizin (DGIIN) zur zielgerichteten Einführung

**DOI:** 10.1007/s00063-026-01494-7

**Published:** 2026-09-15

**Authors:** Leo Benning, Markus Haar, Anna Haftenberger, Dario Antweiler, Oliver Steidle, Ralf Möller, Robert Simon Schiff, Sebastian Wolfrum, Mattis Hartwig, Marek Seitz, Georg Widhalm, Sabine Wingen, Hans-Jörg Busch, Guido Michels

**Affiliations:** 1 Zentrum für Notfall- und Rettungsmedizin, Universitätsklinikum Freiburg, Sir-Hans-A.-Krebs-Straße 1, 79106 Freiburg, Deutschland; 2 Medizinische Fakultät, Albert-Ludwigs-Universität Freiburg, Freiburg, Deutschland; 3 Arbeitsgruppe Digitale Medizin und Künstliche Intelligenz in der Akut‑, Notfall- und Intensivmedizin (KIANI), Deutsche Gesellschaft für Internistische Intensivmedizin und Notfallmedizin, Berlin, Deutschland; 4 Klinik für Intensivmedizin, Universitätsklinikum Hamburg-Eppendorf, Hamburg-Eppendorf, Deutschland; 5 Gleiss Lutz Rechtsanwälte Berlin, Berlin, Deutschland; 6 Fraunhofer IAIS, Sankt Augustin, Deutschland; 7 Stabstelle Qualitätsmanagement und klinisches Risikomanagement, Universitätsklinikum Essen, Essen, Deutschland; 8 Institute of Humanities-Centered Artificial Intelligence, Universität Hamburg, Hamburg, Deutschland; 9 Deutsches Forschungszentrum für Künstliche Intelligenz (DFKI), Lübeck, Deutschland; 10 Interdisziplinäre Notaufnahme, Universitätsklinikum Schleswig-Holstein, Lübeck, Deutschland; 11 singularIT GmbH, Lübeck, Deutschland; 12 Projektmanagement Digitalisierung, Krankenhaus der Barmherzigen Brüder Trier, Trier, Deutschland; 13 Klinik für Neurochirurgie, Medizinische Universität Wien, Wien, Österreich; 14 Deutsche Gesellschaft für Internistische Intensiv- und Notfallmedizin e. V. (DGIIN), Berlin, Deutschland; 15 Notfallzentrum, Krankenhaus der Barmherzigen Brüder Trier, Medizincampus Trier der Universitätsmedizin Mainz, Nordallee 1, 54292 Trier, Deutschland

**Keywords:** Notfallmedizin, Intensivmedizin, Patientenversorgung, Digitalisierung, Digitale Transformation, Qualifikation, Digital Health, Interprofessionalität, Prozessintegration, Emergency medicine, Critical care, Patient care, Digitization, Artificial intelligence, Qualification, Digital Health, Interprofessional care delivery, Process integration

## Abstract

In diesem Impulspapier legt die Deutsche Gesellschaft für Internistische Intensiv- und Notfallmedizin e. V. (DGIIN) grundlegende Prinzipien für die zielgerichtete Einführung von Digitalisierung und künstlicher Intelligenz (KI) in der Notfall- und Intensivmedizin dar. Diese Einführung erfordert eine gezielte Qualifizierung aller an der Patientenversorgung beteiligten Berufsgruppen, um technologische Grundlagen, Unsicherheiten, regulatorische Rahmenbedingungen sowie Möglichkeiten und Grenzen medizinischer Anwendungsszenarien in diesen Versorgungsbereichen beurteilen zu können. Zentrales Prinzip für die Entwicklung und Einführung von KI-Anwendungen ist das Co-Development, bei dem klinische Expertise, Wissenschaft, Industrie, Patienten sowie Regulatorik gemeinsam KI-Lösungen entwickeln, um Bedarfsgerechtigkeit, Sicherheit und Praxistauglichkeit zu gewährleisten. Aufgrund unterschiedlicher Versorgungskontexte bedarf es kontextspezifischer Lösungsansätze. Hierbei sind insbesondere die Anforderungen des Medizinprodukterechts, der KI-Verordnung und des Datenschutzes zu berücksichtigen. Die Umsetzung muss eine Integration in bestehende IT-Systeme und verschiedene Dateninfrastrukturen ermöglichen. Die Anwendbarkeit von KI hängt schließlich von organisatorischen Strategien, technischer Interoperabilität und einem aktiv gestalteten, professionellen Kontext ab, sodass KI-Anwendungen in zeitkritischen, medizinischen Situationen wie z. B. auf Intensivstationen und Notaufnahmen verantwortungsvoll, nachvollziehbar und wirksam in Entscheidungen eingebunden werden können.

## Abstract (en)

In this position paper, the German Society for Internal Intensive Care and Emergency Medicine e. V. (Deutsche Gesellschaft für Internistische Intensivmedizin und Notfallmedizin, DGIIN) sets out its principles for the introduction of digitization and artificial intelligence (AI) into emergency and intensive care medicine. A prerequisite for their introduction is a targeted qualification for physicians and healthcare professions so that they can assess technological fundamentals, uncertainties, and regulatory frameworks, as well as the possibilities and limitations of medical use cases. The central principle for the development and introduction of digitization and AI applications is co-development, in which clinical expertise, science, industry, and regulators jointly develop digitization and AI solutions to ensure that they meet clinical needs, are safe, and are practical to use. Due to different care delivery contexts, context-specific solutions are required. In particular, the requirements of medical device regulations, AI regulations, and data protection must be taken into account. Implementation must enable integration into existing IT systems and interoperable data infrastructures. Ultimately, the applicability of AI depends on organizational implementation strategies, technical interoperability standards, and an actively designed professional context so that digitization and AI applications can be integrated into clinical decision-making in a responsible, transparent, and effective manner—all instrumental features for their use in time-critical medical situations, e.g., in intensive care units and emergency departments.

## Einleitung

Das Gesundheitswesen befindet sich in einem tiefgreifenden Transformationsprozess. Steigender Versorgungsbedarf, zunehmende Komplexität sowie wachsende regulatorische und ökonomische Anforderungen treffen auf begrenzte personelle und finanzielle Ressourcen. Der Fachkräftemangel gilt laut der Organisation for Economic Cooperation and Development (OECD) in nahezu allen Mitgliedsstaaten als zentrales Risiko für die Versorgungssicherheit [1]. Zugleich steigen die Gesundheitsausgaben in einem Maße, das die langfristige Tragfähigkeit der öffentlichen Finanzierung unter Druck setzt [2] – in Deutschland insbesondere durch eine hohe Inanspruchnahme von Gesundheitsleistungen [3].

Diese Entwicklungen betreffen die hochkomplexe Notfall- und Intensivmedizin in besonderem Maße. Ihre Versorgungsaufträge sind interprofessionell, ressourcenintensiv und erfordern hochspezialisierte, medizinische Expertise. Entscheidungen müssen häufig unter Zeitdruck und auf Basis unvollständiger Informationen getroffen werden. Gleichzeitig entstehen große Mengen klinischer Daten, z. B. aus Monitoring-Systemen, bildgebender sowie Labordiagnostik, elektronischen Patientenakten und administrativen Systemen.

Angesichts des wachsenden Ungleichgewichts zwischen Versorgungsbedarf und verfügbaren Kapazitäten gewinnen KI-Lösungen und digitale Technologien zunehmend an strategischer Bedeutung. Sie bieten die Möglichkeit, diese Daten systematisch auszuwerten und medizinische Entscheidungsprozesse zu unterstützen. Ziel ist dabei nicht das Ersetzen fachlicher Expertise der Berufsgruppen, sondern deren gezielte Unterstützung. Voraussetzung für eine erfolgreiche Integration solcher Technologien ist ein strukturiertes Vorgehen, das medizinische, organisatorische, technische und regulatorische Aspekte gleichermaßen berücksichtigt.

Die Deutsche Gesellschaft für Internistische Intensiv- und Notfallmedizin e. V. (DGIIN) sieht in der gezielten Nutzbarmachung von Digitalisierung und künstlicher Intelligenz (KI) in diesen Versorgungsbereichen einen zentralen Ansatzpunkt, um die Leistungsfähigkeit und Zukunftsfähigkeit in der Intensiv- und Notfallversorgung weiter zu sichern. Diese Arbeit soll einen diskursiven Rahmen und eine Priorisierungslogik anbieten, um diese Potenziale in der Notfall- und Intensivmedizin zu realisieren.

Hierzu müssen zunächst alle an der Versorgung beteiligten Berufsgruppen *Qualifikationen* erwerben, um eine aktive und gestaltende Rolle in diesem Feld einnehmen und KI-Systeme praxisrelevant, sicher und patientenzentriert entwickeln und anwenden zu können. Durch geeignete Qualifikationsmaßnahmen sollten sich auch die an der Intensiv- und Notfallversorgung beteiligten Professionen damit zunehmend zu einer gestaltenden Instanz an der Schnittstelle von Gesundheitsversorgung, technologischer Innovation und regulatorischem bzw. gesundheitspolitischem Rahmen entwickeln [4].

Eine ausreichende Qualifikation ermöglicht das sog. *Co-Development*, die gemeinsame Entwicklung von KI-Systemen durch klinische Anwender, Wissenschaft, Industrie und Regulatoren [5]. Nur ein solches kooperatives Entwicklungsmodell kann sicherstellen, dass KI-Anwendungen nicht an den Bedürfnissen der klinischen Praxis vorbeientwickelt werden. Für die Notfall- und Intensivmedizin bedeutet dies: KI-Anwendungen dürfen nicht als fertiges Produkt „von außen“ eingeführt werden, sondern sollten innerhalb des Versorgungskontextes entstehen, um klinisch relevant, sicher und vertrauenswürdig zu sein.

Über das Co-Development hinaus erfordert die erfolgreiche Implementierung eine *Kontextspezifität*, die nicht nur den klinischen, sondern auch den regulatorischen und den Systemkontext berücksichtigt. Eine „Off-the-shelf-Implementierung“ (unveränderte Übernahme aus einem anderen Kontext) von in anderen Gesundheitssystemen entwickelten Systemen in das deutsche oder europäische Gesundheitssystem geht mit erheblichen Risiken einher: Versorgungskontexte, regulatorische Vorgaben und der rechtliche Rahmen unterscheiden sich erheblich [6]. So sind Digitalisierungs- und KI-Anwendungen mit medizinischer Zweckbestimmung als „Software as a Medical Device (SaMD)“ einzustufen und unterliegen der europäischen KI-Verordnung (KI-VO). Im Gegensatz zu konventionellen Medizinprodukten sind sie dynamisch und können sich durch kontinuierliches Training verändern. Diese Eigenschaft muss in existierende rechtliche und regulatorische Rahmenbedingungen eingebettet werden. Gesetzgeberische Initiativen wie der European Health Data Space (EHDS) oder das Gesundheitsdatennutzungsgesetz (GDNG) können in diesem Rahmen entscheidende Impulse für eine verantwortungsvolle und interoperable Nutzung von Gesundheitsdaten setzen.

Abschließend ist die *Anwendbarkeit* von KI-Ergebnissen entscheidend. Selbst wenn Modelle valide Vorhersagen liefern, bleibt häufig unklar, wie diese in konkrete klinische Handlungen übersetzt werden können [7]. Der Abstand zwischen praktischer Anwendung bzw. algorithmischer Ausgabe und ärztlicher Entscheidung muss daher nicht nur organisatorisch und technisch, sondern auch hinsichtlich Kompetenz- und Kulturfaktoren überbrückt werden [7]. Dieser Aspekt rückt die Integration von KI in bestehende Abläufe und Entscheidungsstrukturen in den Mittelpunkt.

## Abgrenzung und Zielsetzung des Impulspapiers

**Abb. 1:**
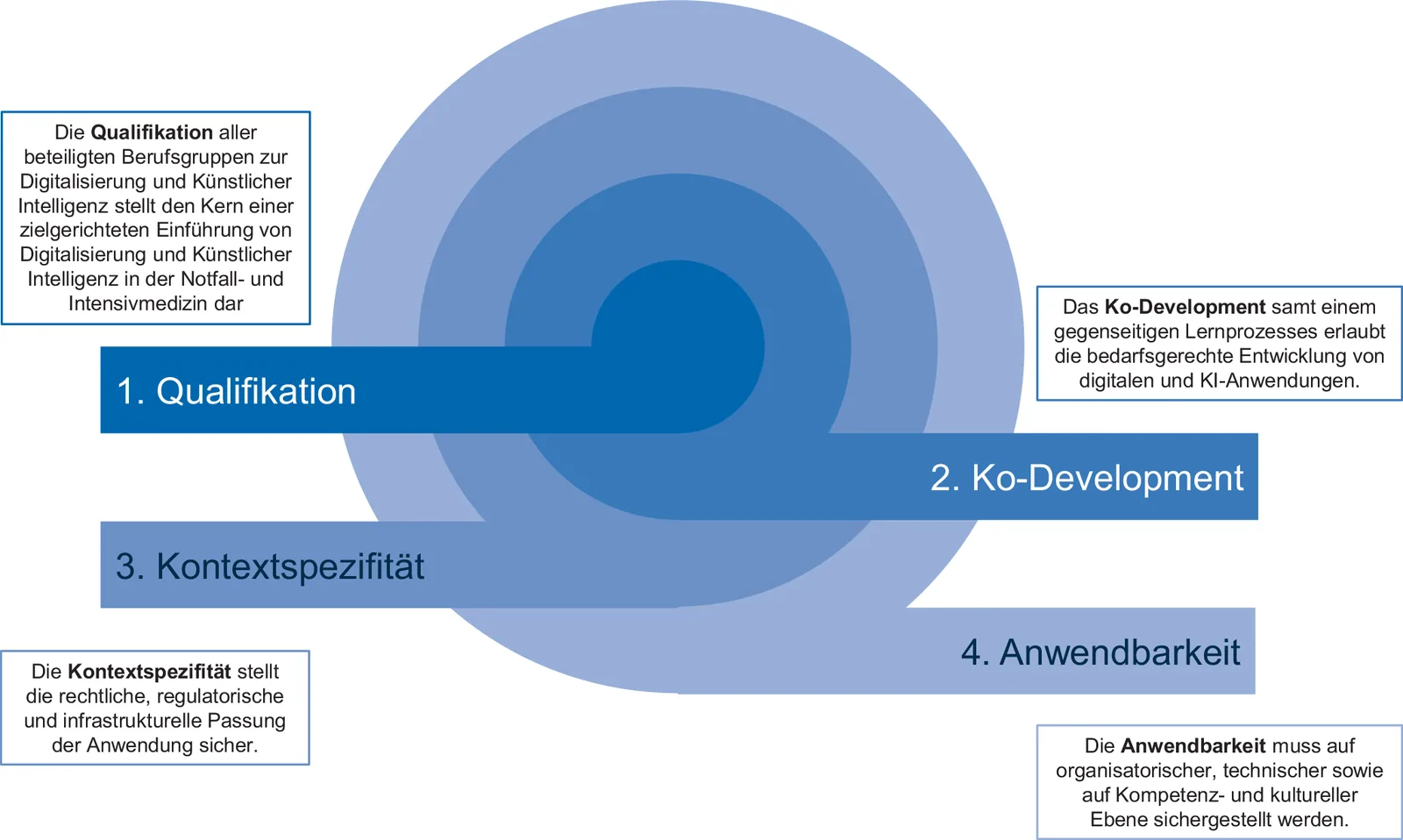
Implementierungsmodell für die Einbettung von Digitalisierung und künstlicher Intelligenz in der klinischen Anwendung der Notfall- und Intensivversorgung

Das vorliegende Impulspapier der DGIIN möchte im Spannungsfeld von technologischer Innovation, klinischer Praxis und gesundheitspolitischer Steuerung Leitgedanken aufzeigen, die für eine verantwortungsvolle Einführung und Entwicklung von digitalen Technologien und KI in der Notfall- und Intensivversorgung dienen können. Es ist aus der Arbeitsgruppe Digitale Medizin und künstliche Intelligenz der DGIIN aus der internen Synthese etablierter Implementierungsstandards entstanden, die gemeinsam mit beteiligten Experten aus den Rechts‑, Daten- und Implementierungswissenschaften auf den Versorgungskontext der Notfall- und Intensivmedizin angepasst wurden. Alle konzeptionellen Elemente wurden durch einen nichtformalisierten, internen Konsensprozess definiert und durch die Arbeitsgruppensprecher in diesem Papier zusammengeführt. Ein besonderer Schwerpunkt liegt auf den oben genannten vier Bereichen Qualifikation, Co-Development, Kontextspezifität und Anwendbarkeit der Technologien, die in Abb. 1 schematisch dargestellt sind. Der Schwerpunkt ist dabei konzeptionell zu verstehen, auf Prinzipien beschränkt und umfasst keine systematische Evidenzsynthese einzelner bereits evaluierter Digitalisierungs- oder KI-Anwendungen, die in der gegenwärtigen Dynamik der technologischen Entwicklung kaum erschöpfend dargestellt werden kann. In Abgrenzung zu Digitalisierungs- und KI-Anwendungen für den klinisch-praktischen Einsatz existieren zudem oftmals Anwendungen mit einem primär administrativen Fokus, die beispielsweise auf eine vereinfachte Dokumentation abzielen [8] oder die dringend notwendige Anpassung von elektronischen Arbeitsumgebungen an klinische Arbeitsabläufe adressieren [9]. Obgleich die Folgeeffekte solcher Anwendungen (z. B. weniger „cognitive burden“ durch intuitive Bedienbarkeit oder höhere Produktivität durch weniger Dokumentationslast) ebenfalls auf die klinische Praxis wirken können, haben sie nur mittelbar Einfluss auf die intensiv- und notfallmedizinische Patientenversorgung und sind deshalb nicht der Fokus dieser Arbeit.

## Qualifikation der Berufsgruppen

Die erfolgreiche Nutzung von digitalen Technologien und KI-Anwendungen auf Intensivstationen sowie in Notaufnahmen setzt ein grundlegendes Verständnis ihrer Funktionsweise, ihrer Möglichkeiten sowie ihrer Grenzen voraus. Medizinisches Personal muss in der Lage sein, Ergebnisse digitaler Systeme kritisch zu bewerten und in klinische Entscheidungsprozesse einzuordnen.

Um alle Berufsgruppen in der Intensiv- und Notfallversorgung in die Rolle zu versetzen, der technologischen Entwicklung von KI-Anwendungen nicht nur zu folgen, sondern diese auch aktiv zu gestalten, ist die gezielte Aus‑, Fort- und Weiterbildung die grundlegende Voraussetzung. Es geht dabei nicht darum, die Berufsgruppen zu Datenwissenschaftlern auszubilden, sondern ein grundlegendes Verständnis darüber zu vermitteln, wie klinische Daten in digitalen Modellen verarbeitet werden und welche Faktoren die Qualität algorithmischer Vorhersagen beeinflussen können. Hierzu gehören beispielsweise die probabilistische Natur von KI-Modellen, mögliche Verzerrungen in Datensätzen sowie Veränderungen von Datenstrukturen im Zeitverlauf. Kenntnisse von künstlicher Intelligenz sollten als Bestandteil der entsprechenden Ausbildungsberufe und Fachweiterbildungen integriert, aber auch aufgaben- und kontextspezifisch in der klinischen Praxis ermöglicht werden. Der Fokus dieser Qualifikationsmaßnahmen sollte auf der Entwicklung einer Querschnittskompetenz liegen, die die Arbeit an den Schnittstellen zwischen medizinischer und technologischer Fachexpertise überbrücken kann und gleichzeitig identifiziert, welche Tätigkeiten zur Vermeidung eines sog. De-Skilling (Verlust von Kompetenzen) von Fachpersonal nicht oder nicht ausschließlich durch technologische Lösungen unterstützt oder übernommen werden sollten. Hierzu halten die Autoren die nachfolgend beschriebene Kompetenzarchitektur von technologischen Grundlagen bis hin zu regulatorischen Rahmenbedingungen für geeignet.

### Grundlagen der Informationsrepräsentation und algorithmische Konzepte

Ärzte und weitere Berufsgruppen in der Intensiv- und Notfallversorgung können nicht flächendeckend mit einer dualen Qualifikation aus fachlicher Kompetenz und einer datenwissenschaftlichen Ausbildung ausgebildet werden. Gerade in den hochspezialisierten Gebieten der Notfall- und Intensivversorgung kann der Kompromiss zwischen diesen Expertisen zu einem „Kompromiss zum Generalisten“ führen, der für die Versorgungsqualität von Patienten nachteilig sein kann. Sehr wohl aber kann ein Verständnis vermittelt werden, wie klinische Daten in verschiedenen Modellarten abgebildet und analysiert werden können.

### Anwendungsfelder und Lösungsräume

Ein Verständnis von Informationsrepräsentation und algorithmischen Konzepten schafft die Grundlage, um die Anwendbarkeit von Digitalisierung und KI-Anwendungen zu beurteilen. Gleichzeitig ermöglicht die Expertise der Berufsgruppen die Einschätzung, ob ein fortschrittlicher Lösungsansatz durch eine KI-Anwendung einen Mehrwert in der Versorgung schaffen kann, ob einfache, regelbasierte Lösungsansätze bei weniger Implementierungsaufwand eine vergleichbare Leistungsfähigkeit entwickeln können oder ob durch die Implementierung Risiken und mögliche negative Folgeeffekte resultieren können. Insbesondere die Notwendigkeit des Kompetenzerhalts der Berufsgruppen erfordert eine kritische Perspektive darauf, welche Aufgaben oder Tätigkeiten zwingend bzw. prioritär in der alleinigen Verantwortung der betreffenden Berufsgruppen verbleiben sollten oder wo ein „automation bias“ das Risiko für das unreflektierte Vertrauen in algorithmische Empfehlungen steigert. Abschließend ist die Expertise in der klinischen Anwendung der beteiligten Berufsgruppen zentral, um die Einbettung von geeigneten Digitalisierungs- und KI-Anwendungen in die Behandlungsabläufe fundiert beurteilen und mitgestalten zu können.

### Unsicherheit, Fehlermodi und Modellgrenzen

Durch die Kenntnis des jeweiligen notfall- oder intensivmedizinischen Versorgungskontextes kann der kritische Pfad einer technologischen Lösung präzise beschrieben werden. Durch ein Verständnis der probabilistischen Natur von KI-Anwendungen kann die Abwägung einer vertretbaren Unsicherheit kontext- und anwendungsspezifisch erfolgen. Hierzu ist ein fundierter Überblick über Datenheterogenität, Bias-Kategorien, Overfitting (Überanpassung auf Trainingsdaten), Datendrift (schleichende Veränderung von Mustern in Real-World-Daten im Vergleich zu Trainingsdaten) und Halluzinationen (Erzeugung sachlich falscher, jedoch semantisch plausibler Ausgaben) entscheidend. Nur mit diesen Kenntnissen kann die Beurteilung der klinischen Eignung einer KI-Anwendung erfolgen.

### Evaluierung und Dokumentationspflicht

Während die obigen Aspekte die Plausibilisierung einer KI-Anwendung ermöglichen, ist eine formalisierte Evaluierung ein entscheidender Schritt, um eine KI-Anwendung in den Versorgungsalltag zu integrieren. Neben Validierungsstrategien und den Voraussetzungen für ein Monitoring der Anwendung, ggf. erforderliche Rekalibrierungen und eine Nachvollziehbarkeit von ggf. notwendigen Anpassungen durch einen sog. Audit Trail (Mechanismus zur Nachverfolgung von Änderungen) sind auch Wirksamkeit und Sicherheit der Anwendung nach einem präspezifizierten Prüfschema zu erheben. Diese Schritte sind Grundlage für eine rechtssichere Anwendung und ermöglichen die Auswahl sicherer Anwendungen für die Patientenversorgung in einem Hochrisikosetting.

### Ethik, Recht und Governance

Über die medizinisch-wissenschaftliche Evaluierung hinaus sind ethische und rechtliche Rahmenbedingungen von zentraler Bedeutung, um KI-Anwendungen in die intensiv- und notfallmedizinische Versorgung zu überführen. In diesem Kompetenzbereich sind Fragen zu der Repräsentativität der verwendeten Daten, dem Stellenwert von KI-Anwendungen in der klinischen Entscheidungsfindung, der Gerechtigkeit von und Gleichbehandlung durch Digitalisierungs- und KI-Anwendung und Erwägungen zur Haftung zu adressieren.

## Co-Development

Gelingt die Entwicklung der oben genannten Querschnittskompetenz, können gleichermaßen leistungsfähige wie auch bedarfsgerechte Digitalisierungs- bzw. KI-Anwendungen gezielt für die Intensiv- und Notfallversorgung entwickelt und implementiert werden. Internationaler „best practice“ entsprechend, können durch das Co-Development klinische Anwender, Wissenschaftler und Industrieakteure wirksam zusammenarbeiten. Hierzu schlägt die Arbeitsgruppe Digitale Medizin und künstliche Intelligenz der DGIIN ein strukturierendes Rahmenwerk zur Projektentwicklung und Implementierung vor. Als Fallbeispiel eines erfolgreichen Co-Development-Ansatzes wird im letzten Abschnitt auf das Assistenzsystem zur Prozessoptimierung in der Notaufnahme (APONA) eingegangen.

### Methodischer Rahmen

Ein strukturierendes Rahmenwerk kann als Plattform für das Co-Development einer Digital- oder KI-Anwendung dienen und durch eine nachvollziehbare Dokumentation die erforderliche Transparenz schaffen, für welchen Zweck ein Modell entwickelt wird, auf welchen technologischen Prinzipien es basiert, in welchen Versorgungskontexten es Verwendung finden soll, welche Grenzen die Anwendung hat und welche Maßnahmen erforderlich sind, um Fehlinterpretationen oder sicherheitsrelevante Zwischenfälle zu vermeiden. Für die Hochrisikoversorgungsbereiche Intensiv- und Notfallmedizin hält die DGIIN die Entwicklung eines entsprechenden Rahmenwerks für essenziell und sieht die Möglichkeit, dieses, angelehnt an bereits bestehende Rahmenwerke, zu entwickeln. Ein Beispiel für ein solches Rahmenwerk ist die Applied Model Card der Coalition for Health AI (CHAI), die sowohl als Kommunikationsinstrument zwischen klinischen Anwendenden, Entwickelnden und Entscheidungsträgern als auch als Grundlage für die erforderliche Governance und das Monitoring der Anwendung im Betrieb fungiert [10]. Eine ähnliche Struktur hat das Rahmenwerk European Responsible AI Development (EURAID), das einen besonderen Fokus auf europäische Werte legt [11]. In Anlehnung an die obigen Beispiele hält die DGIIN einige Kategorien eines strukturierenden Rahmenwerks für Digitalisierungs- und KI-Anwendungen in der Intensiv- und Notfallversorgung für wesentlich, die im Folgenden aufgeführt werden.

#### Zweckmäßiger und „Out-of-scope-Gebrauch“

Der zweckmäßige Gebrauch einer Digitalisierungs- oder KI-Anwendung muss eindeutig definiert werden. Gleichzeitig muss dieser ausdrücklich von einer nichtintendierten Nutzung abgegrenzt werden, um den regulatorischen Rahmen zu definieren und Missbrauch bzw. eine irrtümliche Anwendung zu vermeiden.

#### Nutzende

Nach der Beschreibung von Zweck und Funktion geht die Applied Model Card auf die Nutzenden der KI-Anwendung ein. Hier muss zwischen primären Nutzenden (u. a. Ärzte, Gesundheitsfachpersonal, technische Mitarbeitende und Assistenzberufe) und sekundär Nutzenden (z. B. Entwickelnde, Wissenschaftler, administratives Personal) unterschieden werden. Zudem muss die Rolle der Nutzenden samt Fähigkeiten und Verantwortlichkeiten beschrieben werden. Ist beispielsweise medizinische Fachkenntnis erforderlich, um eine therapeutische Konsequenz aus einer Ausgabe der KI-Anwendung zu interpretieren, muss dies konkret definiert werden.

#### Modellbeschreibung

Die Erläuterung der technischen Eigenschaften sollte die verwendeten Modelle, Algorithmen, verwendete Datenformate für Input und Output, ggf. erforderliche Vorverarbeitungsschritte und technisch bedingte Limitationen der Anwendung umfassen und den klinischen Anwendungsfall berücksichtigen.

#### Daten

In diesem Abschnitt wird die Datenbasis samt Herkunft, Umfang und möglichen Einschränkungen, beispielsweise hinsichtlich der zeitlichen oder medikolegalen Verfügbarkeit von Daten erläutert. Aus dieser Beschreibung können resultierende Verzerrungen und Bias abgeleitet und bewertet werden.

#### Leistungsevaluation

Die Evaluierung der Anwendung sollte a priori beschrieben und im Falle einer In-silico-Evaluierung, z. B. auf Basis etablierter Datenbanken [12–14], anschließend im realen Versorgungskontext überprüft werden. Geeignete Leistungsparameter (Key Performance Indicators) müssen für Patienten und für die Behandlung relevante Ergebnisse abbilden und dürfen sich nicht auf technische Prozesskennzahlen beschränken. Eine Definition einheitlicher Qualitätsindikatoren und Kennzahlen im Sinne eines Qualitätsmanagements für digitale und KI-Systeme/Produkte sollte angestrebt werden und ist entscheidend, um einen tatsächlichen Versorgungseffekt von einer technischen Wirksamkeitsillusion abzugrenzen.

#### Deployment

Digitalisierungs- und KI-Anwendungen können nur in seltenen Fällen ohne Integration in eine bestehende Systemlandschaft implementiert werden. Ein erfolgreiches Co-Development muss daher frühzeitig erforderliche Schnittstellen in lokale Krankenhausinformationssysteme (KIS), Radiologieinformationssysteme (RIS) oder andere IT-Systeme, (inter-)operable Datenformate und potenzielle Implikationen einer standortübergreifenden Nutzung von Daten berücksichtigen. An diesem Punkt sind auch ökonomische Aspekte zu beachten (z. B. Implementierungskosten, Vergütungsmodalitäten, Compliance und Haftungsaspekte), die eine Implementierung entscheidend beeinflussen können.

#### Überwachung und Instandhaltung

Da KI-Anwendungen dynamische Systeme darstellen, ist nach dem Deployment die Entwicklung nicht abgeschlossen. Die anschließende Überwachung im laufenden Betrieb muss Datendrifts und mögliche Leistungsabfälle erkennen [15]. Hierzu sollten ein leistungsfähiger Co-Development-Prozess festgelegt und veränderte Leistungsparameter dokumentiert werden, welche Schwellen ein erneutes Training zugrunde liegender Modelle auslösen und wie ggf. vorgesehene Updates in den Co-Development-Prozess aufgenommen werden können.

#### Ethische und Sicherheitserwägungen

Die in den obigen Abschnitten identifizierten Risiken müssen kritisch evaluiert und insbesondere hinsichtlich der Relevanz für die Patientensicherheit in der Intensiv- und Notfallversorgung, aber auch hinsichtlich möglicher haftungsrechtlicher Implikationen für die Nutzenden bewertet werden. In diesem Schritt können auch Empfehlungen formuliert werden, wie mit Ausgaben der betreffenden KI-Anwendung umzugehen ist. Ein iteratives Vorgehen, das Bewertungen aus ethischen und Sicherheitserwägungen aufgreift, um oben genannte Passagen anzupassen, kann die Qualität des Co-Development-Prozesses steigern.

### Anwendungskontext

**Abb. 2:**
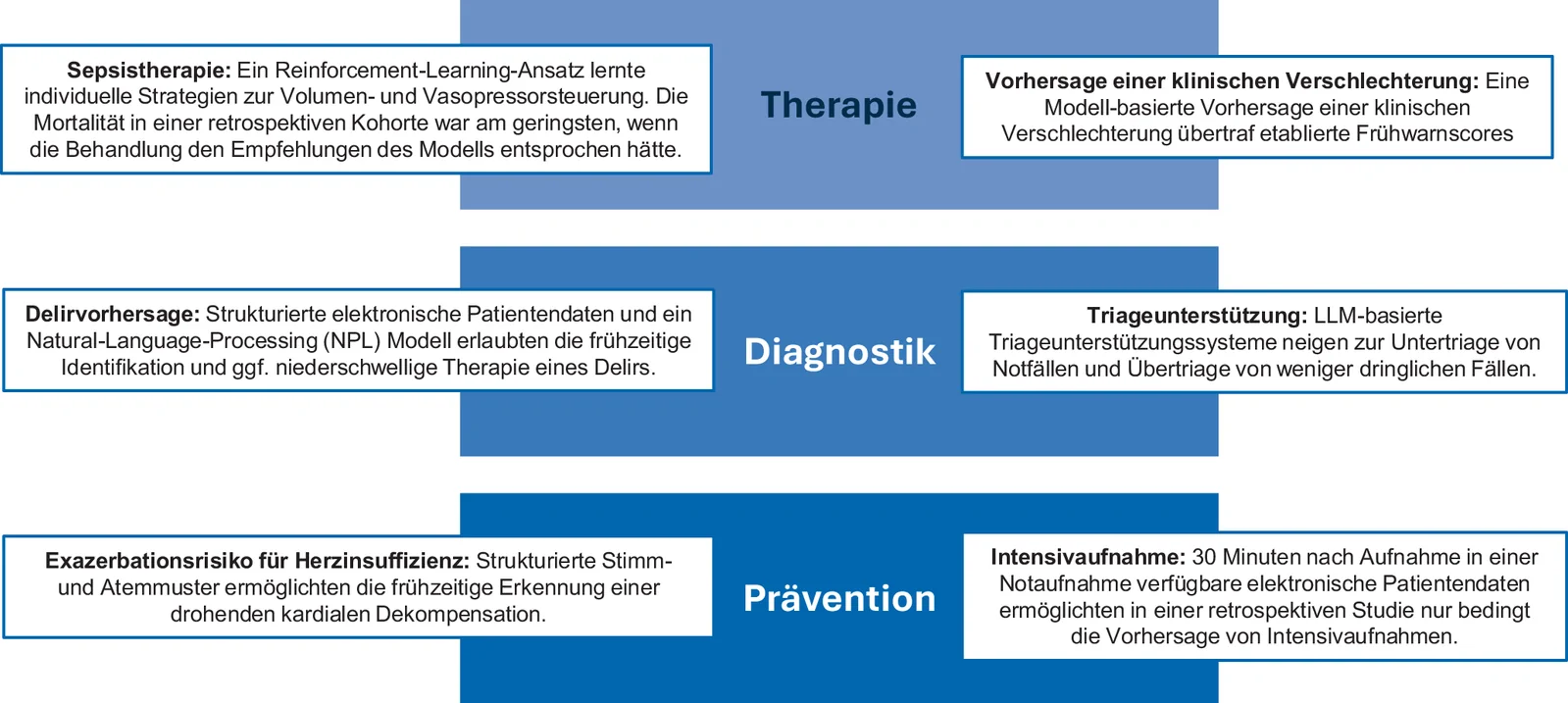
Illustrative Darstellung von Digitalisierungs- und KI-Anwendungen in typischen Anwendungsfeldern der Notfall- und Intensivmedizin. Sepsistherapie: 16; Vorhersage einer klinischen Verschlechterung: 17; Delirvorhersage: 18; Triageunterstützung: 19 ; Exazerbationsrisiko für Herzinsuffizienz: 20; Intensivaufnahme: 21.

Die Anwendungsfelder von Digitalisierungs- und KI-Lösungen in der Notfall- und Intensivmedizin sind vielfältig und eine erschöpfende Darstellung bisheriger Implementierungsbeispiele ist angesichts der raschen technologischen Entwicklung nicht zielführend. Zur Illustration führt Abb. 2 einige Beispiele zusammen, die notfall- oder intensivmedizinisch relevant sind, jedoch allesamt keine vollständige Implementierung durchlaufen und somit auch nicht in der klinischen Anwendung etabliert sind. Nachfolgend wird das Fallbeispiel des Assistenzsystems zur Prozessoptimierung in der Notaufnahme (APONA) ausführlicher beschrieben, das auch außerhalb einer wissenschaftlichen Evaluierung konkrete Schritte zur perspektivischen Implementierung in der Patientenversorgung unternommen hat.

### Anwendungsbeispiel: Assistenzsystem zur Prozessoptimierung in der Notaufnahme (APONA)

Der Ansatz, durch eine Analyse von Datenströmen aus der Notaufnahme die dortigen Prozesse mit patientenspezifischen Vorhersagen in Echtzeit zu optimieren, verspricht kürzere Wartezeiten für Patienten, eine Kostensenkung durch besser gesteuerte Prozesse im gesamten Krankenhaus und eine Entlastung des medizinischen Personals. Mit einer Darstellung von aktuellen Daten der Notaufnahme zu geschätzten relevanten Größen – wie Bettenbedarf in Fachabteilungen sowie Dauer des Aufenthalts in der Notaufnahme (bzw. deren Auslastung) – können Prozesse sowohl in der Notaufnahme als auch im gesamten Krankenhaus optimiert werden und erlauben somit, zum Kristallisationskern einer einrichtungsweiten KI-Strategie zu werden. Zur Evaluation der Praktikabilität und Realisierung der genannten Möglichkeiten wurde das Verbundprojekt Assistenzsystem zur Prozessoptimierung in der Notaufnahme (APONA [22]) durch die interdisziplinäre Expertise des Universitätsklinikums Schleswig-Holstein (UKSH), des Deutschen Forschungszentrums für Künstliche Intelligenz (DFKI) und der singularIT GmbH mit finanzieller Unterstützung des Landes Schleswig-Holstein etabliert und auf Basis eines Co-Development-Ansatzes entwickelt. Um die Hypothese zu überprüfen, ob sich durch die genannten Schätzungen ein positiver Effekt in Bezug auf Kostenbilanz und das Patientenwohl erzielen lässt, konnten in APONA Daten von etwa 170.000 Notaufnahmevorgängen (2020–2024) des UKSH analysiert werden. Merkmale aus Verwaltungsaufzeichnungen, Triage und medizinischen Untersuchungen wurden verarbeitet, darunter strukturierte Daten mit kategorialen Werten sowie auch Freitext. Ziel war es, KI-basierte Schätzer zu erzeugen, um i.) zu einem in der Notaufnahme eintreffenden Patienten die Krankenhausaufnahme (binär), ii.) die nächste Fachabteilung (14 Klassen) sowie iii.) weitere Parameter, wie z. B. Notaufnahmeauslastungswerte, zu prognostizieren. So sind neben technischen Leistungskennzahlen auch unmittelbar versorgungsbezogene Indikatoren verfügbar. Während zunächst nur Verwaltungsdaten bekannt sind, kommen nach einer Triage-Phase weitere Informationen hinzu, sodass die Schätzer belastbarer werden. Die Schätzwerte nach der medizinischen Untersuchung waren von ausreichender Qualität, sodass eine Anwendung in der Praxis im UKSH angestrebt wird, sobald die Anwendbarkeit nach den unten genannten Kriterien etabliert werden kann. Die Genauigkeit der Bestimmung der besten drei nächsten Fachabteilungen bedingt, dass in den meisten Fällen die richtige Abteilung unter den drei besten Vorschlägen der Auswahllisten zu finden ist. Für die praktische Bettenverwaltung sind solche Ranglisten oft ausreichend, da sie es den Koordinierungsteams ermöglichen, bereits zu Beginn des Patientenaufenthalts mehrere mögliche Stationen in Betracht zu ziehen. Dies reduziert Transferverzögerungen und verbessert den Patientenfluss im gesamten Krankenhaus [23, 24]. Wesentliche Aufgaben des Projekts bestanden in der akribischen Organisation der systematischen Datenerfassung, was den Stellenwert der Kontextspezifität unterstreicht. Der Aufwand für das Einrichten von Trainingssystemen für Schätzer war überschaubar. Beste Ergebnisse wurden durch bekannte Boosting-Verfahren erzielt (hier: CatBoost, ein Verfahren, um kategoriale Werte in die Schätzungen einzubeziehen), die mehrere Lernalgorithmen kombinieren, um in der schrittweisen Kombination ein leistungsfähigeres Lernmodell abzubilden. Freitextfelder in den Daten (z. B. Grund für den Besuch, Diagnose, Anamnese) wurden durch Einbettungsverfahren (aus Large Language Models) vorverarbeitet und in für CatBoost verarbeitbare Werte transformiert. Auch die Infrastruktur für die Bereitstellung der erzeugten Schätzer zur Anwendung in der UKSH-Infrastruktur in Bezug auf periodisch neu erfasste Real-World-Daten in der Notaufnahme stellt einen wichtigen Arbeitspunkt dar, der nach den Erfahrungen im Projekt APONA nicht unterschätzt werden sollte und eine wesentliche Grundlage für eine kontinuierliche Überprüfung der Leistungsfähigkeit der Anwendung im klinischen Alltag darstellt. Schätzerbasierte Systeme mit guten Ergebnissen, wie sie in APONA erreicht wurden, müssen sich in der realen Welt bewähren. Daher wurde in dem Projekt ein konkretes interaktives System zur Analyse von aktuell erhobenen Daten und der Darstellung der Ergebnisse entwickelt, das in der Notaufnahme des UKSH zur Evaluation zum Einsatz kommt, um zu untersuchen, wie Prozesse der Notaufnahme durch ein solches System beeinflusst werden. Perspektivisch ist zudem die Evaluation hinsichtlich konkreter patientenbezogener Endpunkte vorgesehen.

## Kontextspezifität

### Rechtsrahmen beim Einsatz von KI in der Notfall- und Intensivversorgung

Die Implementierung und Anwendung von Digitalisierung und KI in der Notfall- und Intensivversorgung unterliegen einem komplexen rechtlichen Gefüge. Zentrale Säulen des rechtlichen Rahmens bilden das Medizinprodukte‑, KI-, Arzt- und das Datenschutzrecht. Auch wenn dieser Rechtsrahmen auf den ersten Blick unübersichtlich und eng erscheinen mag, leistet er einen wesentlichen Beitrag zur Gewährleistung von Patientensicherheit, Transparenz und Verantwortlichkeit im Kontext KI-gestützter Entscheidungsprozesse.

#### Medizinprodukterecht und KI-Verordnung.

KI-Systeme, die in der Entscheidungsfindung oder Patientenversorgung in der Notfall- und Intensivversorgung eingesetzt werden und eine medizinische Zweckbestimmung haben, unterliegen als Medizinprodukte der europäischen Medizinprodukteverordnung (MDR) 2017/745/EU. Dies betrifft z. B. Systeme, die bei Diagnosen unterstützen, Risiken berechnen oder therapeutische Empfehlungen generieren. Flankiert wird die MDR durch nationale Gesetze wie das Medizinproduktedurchführungsgesetz (MPDG), das die MDR in nationales Recht umsetzt, und die Medizinproduktebetreiberverordnung (MPBetreibV), die Pflichten für den Betrieb von Medizinprodukten, etwa in Gesundheitseinrichtungen, aufstellt. Neben medizinprodukterechtlichen Vorschriften gilt bei KI-basierten Medizinprodukten die am 1. August 2024 in Kraft getretene KI-VO 2024/1689/EU. Sie nimmt insbesondere Anbieter und Betreiber von KI-Systemen in die Pflicht. Medizinische Einrichtungen übernehmen i. d. R. die Rolle des Betreibers, da sie die KI-Systeme nicht selbst entwickeln oder im eigenen Namen in den Verkehr bringen, sondern diese von externen Anbietern beziehen. Da KI-basierte Medizinprodukte meist „Hochrisiko-KI-Systeme“ sind, unterliegen die Betreiber insbesondere den Pflichten aus Art. 26 KI-VO, wonach u. a. die Aufsicht über die KI und eine hinreichende Qualität der Eingabedaten gewährleistet werden muss. Hochriskante KI-Systeme müssen zudem strenge Anforderungen, etwa hinsichtlich der Transparenz (Art. 13 KI-VO) und technischen Robustheit (Art. 15 KI-VO), erfüllen. Allerdings hat die Europäische Kommission im Rahmen eines umfassenden Reformvorhabens der MDR vorgeschlagen, Medizinprodukte weitgehend aus dem Anwendungsbereich der KI-VO herauszunehmen, um Doppel- und Überregulierung zu vermeiden [25]. Sollte sich dieser Vorschlag im Gesetzgebungsverfahren durchsetzen, würden künftig die meisten Regelungen der KI-VO nicht mehr für KI-Medizinprodukte gelten. Dies würde für Anbieter, aber auch Betreiber von KI-Systemen eine erhebliche regulatorische Entlastung in der Praxis darstellen. Für die Forschung sehen die MDR und KI-VO bereits jetzt spezifische Ausnahmeregelungen vor, um die Entwicklung der KI-Systeme selbst und den Einsatz von KI-basierten Medizinprodukten zu wissenschaftlichen Zwecken zu erleichtern [26].

#### Ärztliche Verantwortung und Haftung.

Der Einsatz von KI-Systemen entbindet Ärzte nicht von ihrer Verantwortung für Diagnose- und Therapieentscheidungen und damit auch nicht von einer möglichen Haftung. Die Nutzung technischer Hilfsmittel, einschließlich KI, kann Bestandteil einer Behandlung sein, ersetzt jedoch nicht das ärztliche Urteil. So kann etwa ein unreflektiertes Vertrauen in die Ergebnisse einer KI haftungsrechtliche Konsequenzen haben. Nicht nur für die Akzeptanz und das Vertrauen in die KI, sondern auch aus haftungsrechtlicher Perspektive ist es daher von besonderer Bedeutung, dass ärztliches Personal die Ausgaben einer KI-Anwendung nachvollziehen kann. Ein besonderes Augenmerk muss deshalb auf potenziell fehlerhaften oder unvollständigen Trainingsdaten, einer begrenzten Generalisierbarkeit von KI-generierten Ergebnissen, Halluzinationen oder einer sachlich unzutreffenden Bezugnahme liegen. Auch Veränderungen der Leistungsfähigkeit im Zeitverlauf bedürfen neben der unter Punkt „Überwachung und Instandhaltung“ thematisierten technischen Überwachung eine klare fachliche Überwachung. Zentral ist an dieser Stelle, dass auch ein hoher Zeitdruck oder eine Stresssituation die ärztliche Verantwortung nicht relativiert. Beim Einsatz von KI-Anwendungen in der Notfallmedizin müssen die Daten aus diesem Grund so aufbereitet sein, dass auch unter widrigen Bedingungen eine fundierte Bewertung durch ärztliches Personal möglich ist. Zudem kann der Einsatz von KI im Behandlungsalltag besondere, ärztliche Aufklärungspflichten mit sich bringen, die im Einzelfall geprüft werden müssen, um Haftungsrisiken zu vermeiden. Denn nach § 630e Abs. 1 Bürgerliches Gesetzbuch muss über alle wesentlichen Umstände der Behandlung aufgeklärt werden, also u. a. über die Art und Durchführung der Behandlung. Diese umfassen auch den Fall, dass KI eingesetzt wird. Stellt der Einsatz von KI eine Neulandmethode dar, muss zudem sowohl auf Behandlungsalternativen als auch die Möglichkeit bisher noch unbekannter Risiken hingewiesen werden.

#### Datenschutz.

Werden digitale bzw. KI-Anwendungen in der Notfall- und Intensivversorgung eingesetzt, werden zwangsläufig sensible Gesundheitsdaten verarbeitet. Nach der Datenschutzgrundverordnung (DSGVO) 2016/679/EU dürfen Gesundheitsdaten nur verarbeitet werden, wenn einer der Tatbestände des Art. 9 Abs. 2 DSGVO (z. B. Einwilligung, medizinische Diagnostik, Versorgung oder Behandlung, Schutz lebenswichtiger Interessen) erfüllt ist. Krankenhausträger und an der Versorgung beteiligte Personalgruppen müssen gemäß Art. 5 DSGVO zudem die Grundsätze der Zweckbindung, Datenminimierung, Richtigkeit und Speicherbegrenzung beachten. Sollen Gesundheitsdaten zur Forschung über den originären Behandlungszweck hinaus genutzt werden, ist dies u. a. nach einer informierten Einwilligung des Patienten möglich, deren Einholung u.U. Implikationen für die Integration in Behandlungsabläufe in der Notfall- und Intensivversorgung hat. Darüber hinaus ist die Verarbeitung von Gesundheitsdaten zu wissenschaftlichen Forschungszwecken unter den engen Voraussetzungen des Art. 9 Abs. 2 lit. j DSGVO möglich, die der deutsche Gesetzgeber sowohl auf Bundes- als auch auf Landesebene konkretisiert hat. Vor dem Hintergrund des KI-Trainings gewinnt auch der Aufbau eines europäischen Gesundheitsdatenraums zunehmend an Bedeutung. Der EHDS soll den sicheren und grenzüberschreitenden Zugang zu Gesundheitsdaten in der Europäischen Union erleichtern und so die Nutzung von Gesundheitsdaten für Versorgung, Forschung und Innovation fördern. Für die Notfall- und Intensivversorgung eröffnet der EHDS neue Möglichkeiten, KI-Systeme mit umfangreichen und qualitativ hochwertigen Daten zu trainieren und einzusetzen, wobei zugleich ein hohes Maß an Datenschutz und -sicherheit gewahrt bleibt.

### Systemarchitektur und Infrastruktur

Eine erfolgreiche Integration von KI-Anwendungen erfordert eine leistungsfähige und flexible IT-Architektur. Wesentliche Elemente sind in Tab. 1 aufgeführt.

#### Vorgaben des Qualitätsmanagements in der klinischen Versorgung.

Das einrichtungsinterne Qualitätsmanagement (QM) ist in deutschen Krankenhäusern verpflichtend und somit ein prägendes, kontextspezifisches Merkmal für Digitalisierungsvorhaben und KI-Anwendungen. Im Kontext der oben ausgeführten Herausforderungen des rechtlichen Rahmens und der Datenschutzaspekte werden die technischen bzw. digitalen Chancen für QM bislang nur zögerlich realisiert: Eine aktuelle Umfrage der Gesellschaft für Qualitätsmanagement in der Gesundheitsversorgung (GQMG) zeigt, dass nur 27 % der Teilnehmenden Automatisierungsprozesse häufig nutzen [27]. Dieser Umstand kann im Wesentlichen auf eine unzureichende digitale Kompetenz vieler Krankenhäuser zurückgeführt werden, was den bereits oben hervorgehobenen Stellenwert von Schulung und Weiterentwicklung unterstreicht und bereits von Experten für den Bereich der QM spezifiziert wurde [28]. Unter Realisierung dieser Voraussetzungen kann QM künftig als strategische Schnittstelle zwischen Informationstechnologie (IT), Klinikpersonal, Datenschutz und Geschäftsführung fungieren und als Treiber digitaler Transformation dienen. Dafür ist neben einer technischen Infrastruktur v. a. eine innovationsfreundliche Organisationskultur erforderlich.

#### Integration von KI-Anwendungen in Krankenhausinformationssysteme.

Für eine leistungsfähige Integration von KI-Anwendungen in der Intensiv- und Notfallversorgung ist die Integration in bestehende digitale Infrastrukturen unerlässlich. Das KIS bildet dabei das zentrale Element der IT-Landschaft. Es verwaltet Patientenstammdaten, Laborbefunde, Medikationsinformationen und abrechnungsrelevante Dokumentationen und stellt Schnittstellen zu Subsystemen wie dem Laborinformationssystem (LIS), RIS und Bildarchivierungssystem (Picture Archiving and Communication System, PACS) bereit. Die Integration von KI-Produkten in dieses heterogene Umfeld ist jedoch komplex und gleichzeitig für intensiv- und notfallmedizinische Behandlungsfälle unerlässlich. Über die bereits oben adressierten Aspekte von Datenschutz, Informationssicherheit und regulatorischen Anforderungen gilt es, technische Aspekte wie Datenkompatibilität, Netzwerkanbindung und Systemarchitektur zu berücksichtigen. Aus technischer Perspektive bilden Interoperabilität, standardisierte Schnittstellen und Datenqualität die Grundpfeiler einer erfolgreichen Integration. Moderne Kommunikationsprotokolle, wie z. B. Health Level Seven (HL7), Fast Healthcare Interoperability Resource (FHIR), Digital Imaging and Communications in Medicine (DICOM) und Representational-State-Transfer(REST)-basierte Application Programming Interfaces (APIs), ermöglichen den strukturierten und sicheren Datenaustausch zwischen KIS und KI-Anwendungen (Tab. 1, 2).

**Tab. 1:** Elemente einer Systemarchitektur.

*Rechenleistung und Speicherressourcen*	KI-Modelle, insbesondere im Bereich des Deep Learning, benötigen hohe Graphikprozessor(GPU)-Leistung und schnellen Zugriff auf Trainings- sowie Echtzeitdaten, um eine Anwendung im medizinischen Kontext zu ermöglichen
*Netzwerkkonnektivität*	Der Datenaustausch zwischen KIS, PACS, LIS und KI-Systemen muss über gesicherte und latenzarme Verbindungen erfolgen. VPN-Tunneling und interne Virtual Local Area Network(VLAN)-Segmentierung sind etablierte Maßnahmen zur Absicherung und gewährleisten, dass notwendige Datenströme verlässlich verfügbar sind
*Sicherheitsarchitektur*	Zentrale Aspekte sind Zugriffskontrolle, Verschlüsselung und Audit-Logging (d. h. Aufzeichnung von Zugriffen), sodass nur autorisierte Systeme und Benutzer auf medizinische Daten zugreifen können und eine lückenlose Nachvollziehbarkeit ermöglicht wird. Die Sicherheitsarchitektur muss deshalb bereits bei der Konzeption einer KI-Anwendung berücksichtigt werden
*Betriebsmodell*	Je nach Anwendungsfall kann die KI-Lösung lokal (auch „on-premise“ genannt) oder in zertifizierten Cloud-Umgebungen betrieben werden. Während lokale Systeme Vorteile hinsichtlich Datenschutz und Latenz bieten, ermöglicht der Cloud-Betrieb eine bessere Skalierbarkeit und einfachere Aktualisierung der Modelle

*KIS* Krankenhausinformationssystem, *PACS* Picture Archiving and Communication System, *LIS* Laborinformationssystem, *KI* Künstliche Intelligenz, *VPN *Virtuelles privates Netzwerk

**Tab. 2:** Grundlegende interoperable Datenformate in der Medizin.

*HL7 V2/V3*	Health Level Seven. Etablierter Standard für interne Kommunikationsprozesse in Krankenhäusern. Diese transportieren zentrale Informationen wie Patientendaten, Laboraufträge oder Befunde und sind damit Bestandteil vieler Workflows
*FHIR*	Fast Healthcare Interoperability Resource. Eine moderne, webbasierte Architektur, die Daten über Representational-State-Transfer-Schnittstellen (auch REST-APIs genannt) in JSON- oder XML-Formate austauscht. FHIR entwickelt sich zunehmend zur technischen und semantischen Grundlage zukunftsorientierter KIS-Integrationen – insbesondere dort, wo modulare, interoperable und KI-fähige Systemlandschaften aufgebaut werden sollen
*DICOM*	Digital Imaging and Communications in Medicine. Standard für die Speicherung und Übertragung medizinischer Bilddaten, insbesondere in der Radiologie und Kardiologie

## Anwendbarkeit

Die übergreifende Herausforderung in der Nutzbarmachung der Digitalisierung im Allgemeinen und von KI-Anwendungen im Speziellen liegt in der Einbettung dieser Technologien in die bestehende Gesundheitsinfrastruktur. Technologische Insellösungen erzeugen erhebliche Koordinationskosten, die durch das Gesundheitssystem nicht getragen werden können. Insbesondere für die Intensiv- und Notfallversorgung müssen technologische Lösungen entlang des oftmals interdisziplinär komplexen Behandlungspfades gedacht und implementiert werden. Insellösungen hingegen führen häufig zu einer ausbleibenden Adaptation von Innovation und können sogar den Widerstand beteiligter Akteure zur Folge haben. Deshalb muss die Anwendbarkeit von Digitalisierung und KI organisatorische, technische sowie Kompetenz- und Kulturfaktoren berücksichtigen, deren Berücksichtigung idealerweise in einer transparenten Verantwortungs‑/Zuständigkeitsmatrix sichergestellt werden.

### Organisatorische Faktoren

Eine erfolgreiche Integration von Digitalisierung und KI in der Intensiv- und Notfallversorgung sowie auch für andere Versorgungsbereiche hängt maßgeblich von einer die gesamte Organisation umfassende Strategie ab, die über die reine Technologiebeschaffung hinausgeht. Die Implementierung einer effektiven Digitalisierungs- oder KI-Strategie lässt sich in verschiedene Phasen gliedern, die sicherstellen, dass die Einführung von KI-Systemen systematisch und zielorientiert erfolgt. Diese Aufgabe kann durch ein interdisziplinäres Projektmanagementteam unter Beteiligung der Intensivstationen, der Notaufnahme und ggf. Projektpartnern aus der prähospitalen Notfallmedizin erfüllt werden.

#### Zielbestimmung.

In der ersten Phase wird eine klare Vision für den Einsatz von Digitalisierung oder KI im Krankenhaus im Allgemeinen sowie in der Intensiv- und Notfallversorgung im Speziellen entwickelt. Dies beinhaltet die Identifizierung und Einbeziehung aller relevanten Stakeholder, von der Klinikleitung und den betriebswirtschaftlich verantwortlichen Personen über IT-Spezialisten bis hin zum medizinischen Personal. Workshops, Shadowing-Sitzungen zur Beobachtung klinischer Abläufe und eine umfassende Ist-Analyse der bestehenden Prozesse sind hierbei entscheidende Maßnahmen.

#### Potenzialanalyse.

Anschließend werden potenzielle Anwendungsfälle für KI-Technologien unter medizinischen, organisatorischen und ökonomischen Aspekten analysiert, bewertet und priorisiert. Hierbei kommen verschiedene Methoden zum Einsatz, wie Trendanalysen, eine Überprüfung der KI-Bereitschaft („AI readiness check“), „Use-case-Analysen“ sowie eine Wertschöpfungsketten- und SWOT-Analyse (d. h. strukturierte Betrachtung von Stärken, Schwächen, Chancen und Risiken). Ziel ist es, die vielversprechendsten Einsatzbereiche zu identifizieren und die Machbarkeit zu prüfen [29].

#### Planung.

Auf Basis der Potenzialanalyse werden die Auswirkungen der KI-Einführung aufgezeigt. Es wird ein detaillierter Plan erstellt, der den Aufwand, den erwarteten Erfolg, den Zeitrahmen und die notwendigen Maßnahmen festlegt. Werkzeuge wie die Gap-Analyse (d. h. strukturierte Identifikation von Lösungsräumen bzw. Bedarfen) und interdisziplinäre Workshop-Formate helfen dabei, die Lücken zwischen dem Ist- und dem Soll-Zustand zu schließen. Das oben genannte Co-Development kann auf dieser Ebene in die Entwicklung einer Digitalisierungs- und KI-Strategie eingebunden werden.

#### Qualifikation.

Die erfolgreiche Integration von KI-Systemen steht und fällt mit der Akzeptanz und dem Wissen der Anwender. Daher ist die Qualifikation der Mitarbeitenden von zentraler Bedeutung. Durch verschiedene Elemente, wie Schulungen, Seminare und E‑Learnings, soll die digitale Gesundheitskompetenz gefördert werden [30]. Inhalte können dabei nach obigen Prinzipien zur Qualifikation einen anwendungsbezogenen Fokus oder einen offenen Fokus für die Ermöglichung eines „Bottom-up-Innovationsprozesses“ haben, der Entwicklungs- und Implementierungsschemata ermöglicht.

#### Review.

Eine Digitalisierungs- und KI-Strategie kann angesichts der raschen technologischen Entwicklungen und der sich parallel entfaltenden, regulatorischen Landschaft kein statisches Dokument sein. Daher sollte in regelmäßigen Abständen der Fortschritt der Strategieumsetzung überprüft und bewertet werden, um ggf. Anpassungen vorzunehmen und sicherzustellen, dass die formulierten Ziele erreicht werden. Das in der Zielbestimmung verantwortliche Team sollte als Lenkungsausschuss sowohl die Projektverantwortung als auch die organisatorischen Mittel haben, um die erfolgreiche Implementierung vorzunehmen.

### Technische Faktoren

Für die technische Implementierung sind einige grundlegende Voraussetzungen zu schaffen. Um Abhängigkeiten von einzelnen Herstellern zu vermeiden, wird empfohlen, bei der Beschaffung von Soft- und Hardware auf offene und interoperable Schnittstellen zu achten. Proprietäre Systeme können die Integration neuer Technologien und den Datenaustausch erheblich erschweren. Weiterhin ist auf transparente Lizenzmodelle zu achten, um die finanzielle Belastung gering zu halten. Ein zentraler FHIR-Server in Verbindung mit einem Data Lake ermöglicht einen niederschwelligen Zugriff auf Daten und erleichtert die Anwendungsentwicklung. Grundbaustein für die Integration ist die Nutzung standardisierter Austauschformate, um die Kompatibilität zwischen unterschiedlichen und parallel existierenden Systemen sicherzustellen (Tab. 2).

Die Kombination dieser Standards ermöglicht es, KI-Systeme mit klar strukturierten Eingabedaten zu versorgen und deren Ergebnisse wieder in das KIS einzubetten – etwa als standardisierte Befundobjekte, FHIR-Observation-Ressourcen oder strukturierte Entscheidungsunterstützung. So entsteht ein interoperables Ökosystem, in dem KI nicht als isoliertes Zusatztool, sondern als integraler Bestandteil der klinischen Versorgung wirken kann. Neben diesen fachlichen Austauschstandards spielen REST-basierte Schnittstellen eine zentrale Rolle für die praktische Umsetzung moderner Integrationsarchitekturen. REST-APIs dienen häufig als technisches Bindeglied, über das Systeme Daten abrufen, bereitstellen oder Ereignisse auslösen können. Während klassische HL7-V2/V3-Nachrichten und DICOM-Objekte funktionale Inhalte definieren, beschreibt REST v. a., *wie* diese Inhalte transportiert werden. Besonders im Zusammenspiel mit FHIR, das auf REST-Prinzipien aufsetzt und ihnen eine medizinisch-semantische Struktur verleiht, wird deutlich, wie sich etablierte Standards und moderne Webtechnologien ergänzen: HL7 V2/V3 sorgen für stabile Kernprozesse, DICOM strukturiert komplexe Bilddaten und FHIR über REST bietet eine flexible, interoperable Grundlage für die Integration von KI-Anwendungen. Diese Umgebung ermöglicht es somit, KI-Anwendungen effizient in bestehende Infrastrukturen einzubetten.

### Kompetenz- und Kulturfaktoren

Die Integration auf professioneller Ebene muss den konzeptionellen und semantischen Abstand zwischen der Ausgabe einer Digital- oder KI-Anwendung und dem medizinischen Entscheidungspunkt überbrücken. Während diese Herausforderungen in allen Bereichen der Medizin besteht, ist sie in der Notfall- und Intensivversorgung von besonderer Bedeutung, da in diesen Bereichen häufig zeitkritische Entscheidungen getroffen werden müssen, die oft auf unvollständigen oder fragmentierten Informationen basieren.

#### Urteilsfähigkeit.

Die Besonderheit in der medizinischen Entscheidungsfindung in der Notfall- und Intensivversorgung wird durch KI-Anwendungen nicht aufgelöst. Eine scheinbar präzise Ausgabe kann aber dazu verleiten, dieser unkritisch zu folgen. Dieses Phänomen wird zuweilen als anthropomorphe Projektion bezeichnet und kann zum Fehlschluss führen, dass eine KI-Anwendung eine spezifische fachliche Expertise aufweist. Neben einer ausreichenden Kenntnis der technischen und algorithmischen Grundlagen und der probabilistischen Natur von KI-Anwendungen müssen deshalb die Validierung [31] samt möglicher Verzerrungspotenziale [32] und die Grenzen des zweckmäßigen Gebrauchs, aber auch die einzelnen Ausgaben von KI-Anwendungen die Einschätzung der Unsicherheit zulassen. Für die oft multiparametrischen Ergebnisse von KI-Anwendungen sind Parameter wie Sensitivität, Spezifität, Präzision, Genauigkeit oder F1-Scores oft unzureichend. Es wird empfohlen, Parameter wie eine „confidence accuracy“, die den Abgleich der KI-Ausgabe mit einer empirischen Referenz ermöglicht, heranzuziehen [33]. Nur so kann die Anwendung von KI in der klinischen Entscheidungsfindung verantwortungsvoll gelingen und – nicht zuletzt – auch durch das Behandlungsteam auf Intensivstationen und Notaufnahmen verantwortet werden.

#### Gestaltung von Arbeitsabläufen.

Der Mehrwert einer Digitalisierungsinitiative oder der Einführung einer KI-Anwendung muss durch die Anwender wahrnehmbar sein. Geht die Anwendung mit aufwendigen Datenübertragungen oder manuellen Korrekturen einher, ist eine erfolgreiche Implementierung nahezu unmöglich [34] und das Frustrationspotenzial enorm hoch [35]. Arbeitsabläufe müssen daher nicht nur im Sinne eines „Human-centered AI-Ansatz“ umgestaltet werden [36, 37], sondern in der Implementierung sogar einem expliziten „Clinician-first-Ansatz“, der explizit das Behandlungsteam in den Fokus stellt, folgen. Nur dieser klare Fokus auf die unmittelbar an der Patientenversorgung beteiligten Fachkräfte ermöglicht es, diese nicht zusätzlich durch Digitalisierungs- oder KI-Anwendungen zu belasten.

#### Kultureller und interprofessioneller Rollenwandel.

Die Notfall- und Intensivversorgung ist durch hochgradig teamorientierte, zeitkritische Entscheidungsprozesse geprägt. Digitalisierung und KI verändern dabei nicht nur Informationsflüsse, sondern auch Rollenverständnisse innerhalb des Behandlungsteams [38]. Während KI-Anwendungen zusätzliche Entscheidungsgrundlagen bereitstellen können, ersetzen sie nicht die klinische Urteilskraft, ergänzen diese aber. Der mit diesen Innovationen einhergehende Rollenwandel impliziert jedoch auch, dass klinische Expertise zunehmend die Fähigkeit umfasst, zwischen algorithmisch generierten Empfehlungen und situativem Erfahrungswissen zu vermitteln. Das Kompetenzprofil verschiebt sich damit von einer Wissensautorität hin zu einer kuratorischen Kompetenz, die unterschiedliche Datenquellen kritisch gewichten und integrieren muss [39]. Gleichzeitig muss transparent bleiben, wer welche Beiträge leistet und welche Verantwortung trägt. Klare organisationale sowie Kommunikationsstrukturen sind deshalb zentrale, kulturelle Enabler zur Schaffung eines informierten Vertrauens: Sie verhindern sowohl die Überdelegation an KI-Systeme als auch die pauschale Ablehnung technologischer Unterstützung [40].

## Fazit

Die Deutsche Gesellschaft für Internistische Intensiv- und Notfallmedizin e. V. (DGIIN) sieht in der gezielten Nutzbarmachung von digitalen Technologien und KI in der Intensiv- und Notfallversorgung einen zentralen Ansatzpunkt, um die Leistungs- und Zukunftsfähigkeit in diesen Versorgungsbereichen weiter zu sichern. Derzeit bestehen jedoch regulatorische, haftungsrechtliche, ethische und kulturelle Herausforderungen, die im Sinne einer transparenten, sicheren und nachhaltigen Implementierung von Digitalisierungs- und KI-Anwendungen die technologieoffene Innovationskraft bremsen. Aus diesem Grund stellt die Arbeitsgruppe Digitale Medizin und künstliche Intelligenz in der Akut‑, Notfall- und Intensivmedizin der DGIIN in diesem Impulspapier ein strukturierendes Rahmenwerk in den Vordergrund, das mit den Schritten der i.) Qualifikation von an der Versorgung beteiligten Berufsgruppen, dem ii.) Co-Development in der interprofessionellen Zusammenarbeit zwischen klinischen Anwendern, Wissenschaft, Industrie, Patienten und Regulatoren, einer iii.) Kontextspezifität für den konkreten, klinischen Anwendungsfall und einer iv.) organisationalen, technischen und professionell-kulturellen Anwendbarkeit ein Prüfschema für die erfolgreiche und zielgerichtete Einführung von Digitalisierungs- und KI-Anwendungen in der Notfall- und Intensivversorgung bieten kann.

## Data Availability

Alle dieser Arbeit zugrunde liegenden Daten sind in diesem Artikel enthalten.
